# LINC02418 promotes malignant behaviors in lung adenocarcinoma cells by sponging miR-4677-3p to upregulate KNL1 expression

**DOI:** 10.1186/s12890-020-01229-0

**Published:** 2020-08-14

**Authors:** Tao Wang, Ruiren Zhai, Xiuhua Lv, Ke Wang, Junqing Xu

**Affiliations:** 1 Department of Thoracic Surgery, The Second Affiliated Hospital of Air Force Medical University, Xi’an, 710038 Shaanxi China; 2 Department of Tumor Center, Sunshine Union Hospital, Weifang, 261000 Shandong China; 3 Department of Radiology, Xijing Hospital, Fourth Military Medical University, Xi’an, 710032 Shaanxi China; 4 Department of Radiology, Shenzhen University General Hospital, Shenzhen University Clinical Medical Academy, No.1098 Xueyuan Avenue, Nanshan District, Shenzhen, 518055 Guangdong China

**Keywords:** LINC02418, miR-4677-3p, KNL1, LAD

## Abstract

**Background:**

Lung adenocarcinoma (LAD) is a prevalent type of bronchogenic malignant tumor and one of the most critical factors related to human death. Long noncoding RNAs (lncRNAs) are involved in many complex biological processes and have been emerged as extremely important regulators of various cancers. LINC02418, a novel lncRNA, hasn’t been mentioned in previous studies on cancer development. Therefore, it’s important to define the potential function of LINC02418 in LAD.

**Methods:**

Gene expression was examined by RT-qPCR or western blot. CCK-8, colony formation, TUNEL, and transwell assays were utilized to study the role of LINC02418 in LAD. The interaction of miR-4677-3p with LINC02418 (or KNL1) was verified through luciferase reporter, RIP and RNA pull-down assays.

**Results:**

High expression of LINC02418 was observed in LAD specimens and cells. Downregulation of LINC02418 obstructed the proliferation and motility of LAD cells. Moreover, LINC02418 negatively modulated miR-4677-3p expression and miR-4677-3p overexpression could repress cell proliferation and migration. Moreover, kinetochore scaffold 1 (KNL1) expression was negatively modulated by miR-4677-3p but positively regulated by LINC02418. Furthermore, miR-4677-3p could bind with LINC02418 (or KNL1). Finally, KNL1 overexpression reversed the inhibitory function of LINC02418 deficiency in the malignant behaviors of LAD cells.

**Conclusions:**

LINC02418 contributes to the malignancy in LAD via miR-4677-3p/KNL1 signaling, providing a probable therapeutic direction for LAD.

## Background

As a main subtype of non-small cell lung cancer (NSCLC), lung adenocarcinoma (LAD) is one of the leading causes of cancer-related deaths around the world [1, 2]. Previous studies have identified major pathways involved in LAD development (30–60%), including the activation of the EGFR, KRAS, and ALK signals [3–5]. Although various molecular targeted therapies have been developed, the prognosis of LAD patients is still disappointing [6, 7]. Therefore, defining the molecular mechanisms underlying this fatal disease would be of considerable significance for LAD treatment.

Long non-coding RNAs (lncRNAs) are a subtype of non-coding RNAs (ncRNAs), consisting of RNA over 200 nucleotides in length that are not translated into proteins [8, 9]. Mounting evidence has elucidated a pivotal role of lncRNAs in cancer progression. For example, lncRNA ANCR regulates EZH2 expression to inhibit breast cancer progression [10]. LncRNA LINC00312 expedites cell migration and vasculogenic mimicry in LAD by binding with YBX1 [11]. LncRNA-SNHG1 modulates DNMT1 expression to accelerate the development of gastric cancer [12]. A large number of studies have unmasked that lncRNAs exert critical functions in diverse processes in lung cancer, such as proliferation [13], migration [14] and epithelial-mesenchymal transition (EMT) [15]. Additionally, the involvement of lncRNAs in LAD is also recognized. For instance, lncRNA DGCR5 suppresses the expression of miR-22-3p to promote LAD progression [16]. Galectin-3 activates TLR4/NF-κB signaling pathway to facilitate the development of LAD via upregulating lncRNA-NEAT1 expression [17]. LncRNA MIR31HG overexpression promotes cell proliferation in LAD and associates with poor prognosis [18]. The oncogenic property of some common lncRNAs in LAD has been widely reported, such as LINC00707 [19], MIR31HG [18], OIP5-AS1 [20], MALAT1 [21], etc. Herein, we intended to probe into the biological role of a novel lncRNA in LAD. Using microarray analysis, differentially expressed lncRNAs were identified. The top ten upregulated lncRNAs in LAD samples were chosen for further analysis in LAD cells. LINC02418 was selected to be the research object in current study.

Mechanistically, lncRNAs can interact with microRNAs (miRNAs) to upregulate messenger RNAs (mRNAs) [22, 23], therefore forming a competing endogenous RNA (ceRNA) pathway. Here, bioinformatics analysis and mechanism-based experiments were used to determine the miRNAs that could bind with LINC02418. Similarly, the target mRNAs of miR-4677-3p were identified. In conclusion, this study was designed to investigate whether lncRNA LINC02418 could affect LAD development via regulating its downstream genes.

## Methods

### Cell culture and transfection

Human LAD cell lines (A549, SPC-A1, H1299 and PC-9) and normal lung epithelial cells BEAS-2B were bought from the Cell Bank of the Chinese Academy of Sciences and incubated with Dulbecco’s modified Eagle’s medium (DMEM; Invitrogen Life Technology Inc., Carlsbad, CA), containing 10% fetal bovine serum (FBS) with 5% CO_2_ at 37 °C in humid air. All cell lines were authenticated via STR profiling before using.

The suppression of LINC02418 expression was achieved by sh-LINC02418#1/2. Sh-LINC02418#1 and sh-LINC02418#2 were obtained from GenePharma (Shanghai, China). LINC02418 and KNL1 were overexpressed with pcDNA3.1 vectors (Invitrogen, Carlsbad, USA). MiR-4677-3p mimics were applied to elevate miR-4677-3p expression. MiR-4677-3p mimics and NC mimics were also bought from GenePharma (Shanghai, China). The transfection of above plasmids was conducted by use of Lipofectamine® 2000 agent (Invitrogen).

### Clinical samples collection and microarray analysis

Three pairs of LAD and matched non-cancerous tissue samples were acquired from patients (including two male, one female; two patients < 50 years old, one patient > 50 years old; one patient at stage of I-II; two patients at stage of III-IV) who received operation in the Second Affiliated Hospital of Air Force Medical University. Patients enrolled in this study signed the informed consents. Ethic Committee of the Second Affiliated Hospital of Air Force Medical University has approved sample collection of this research. Microarray analysis was implemented to profile the expression of lncRNAs in LAD. In detail, total RNA was isolated from three pairs of tissues and quantified utilizing NanoDrop 2000 (Thermo, Waltham, MA, USA), followed by quality-checking by use of Agilent Bioanalyzer 2100 (Agilent Technologies, Santa Clara, CA, USA). Subsequently, the GeneChip 3’IVT express kit (Affymetrix, Santa Clara, CA, USA) was utilized to label the qualified RNA samples and then Affymetrix GeneChip Primeview Human cDNA microarray was used for hybridization in line with the manufacturer’s guides. After that, data were analyzed using GeneChip Scanner 3000 (Affymetrix).

### Real-time quantitative polymerase chain reaction (RT-qPCR)

Total RNA was extracted utilizing TRIzol (Invitrogen; Thermo Fisher Scientific, Inc.) and diluted to 200 ng/ml. Complementary DNA (cDNA) synthesis was conducted via applying Taqman Advanced miRNA cDNA Synthesis Kit or Pyrobest DNA Polymerase and M-MLV Reverse Transcriptase (Thermo Fisher Scientific, USA). RT-qPCR was then operated using One Step SYBR® Prime Script™ RT-PCR Kit II (Takara Biotechnology Co., Ltd., Dalian, China) based on the producer’s protocol. Gene expression relative to GAPDH or U6 was assessed using the 2^-ΔΔCt^ method.

### Cell counting kit-8 (CCK-8) assay

Cell proliferation was estimated utilizing the CCK-8 kit (Boster) based on the manufacturer’s requirements. Briefly, cells (1 × 10^3^) were supplemented into 96-well plates. After cell adhesion, each well received 10 μl CCK-8 solution and then cells were further incubated for 1 h at 37 °C. Cell proliferation ability was monitored by detecting absorbance at 450 nm utilizing microplate reader (EL340; Bio-Tek Instruments, Hopkinton, MA, USA) at the indicated time points (0, 24, 48, 72 and 96 h).

### Colony formation assay

Cells were seeded in six-well plates and grown in media with 10% FBS. Two weeks later, the colonies were fixated using methanol and dyed using 0.1% crystal violet for half an hour. Colonies with over 50 cells were counted manually.

### Transwell assay

For invasion estimation, 5 × 10^4^ cells were added onto the upper chambers (BD Biosciences, San Jose, CA, USA) with Matrigel-coating and incubated in DMEM. DMEM supplementing with 10% FBS was put into the bottom chambers. Twenty-four hours later, cotton swabs were used to scrape off cells in the upper chamber. The methanol and 0.5% crystal violet were separately use to fasten and color the cells in the lower chamber. For the migration assays, transfected cells were seeded into the upper chambers with no Matrigel-coating, while other steps were similar to that in invasion assays. Finally, the invaded or migrated cells were counted using an inverted biological microscope (magnification, Å ~ 200).

### Western blot

Cells were lysed by use of RIPA (Beyotime, Shangahi, China) containing protease inhibitor cocktail (Roche, Pleasanton, CA) and phenylmethylsulfonyl fluoride (Roche). Protein samples were then subjected to sodium dodecyl sulfate polyacrylamide gel electrophoresis (SDS-PAGE), followed by transferring onto nitrocellulose (NC) membranes (Sigma-Aldrich). After blocking via 5%-skim milk, the membranes were cultured with primary antibodies (dilution 1:1000) against Bax, Bcl-2, E-cadherin, N-cadherin, MRP2, MRP9, KNL1 (Cell Signaling Technology, Danvers, MA), followed by incubation with secondary antibodies (dilution 1:10000) for an hour at room temperature. β-actin or GAPDH was the loading control. Thereafter, signals were captured with the employment of the ECL chromogenic substrate.

### Terminal-deoxynucleoitidyl transferase mediated nick end labeling (TUNEL) assay

After fixation and permeabilization, cells were processed with dUTP-end labeling (Clontech, Mountain View, CA) and (4′,6-diamidino-2-phenylindole) DAPI, in succession. After that, cells were observed and analyzed under fluorescent microscope (Olympus, Tokyo, Japan).

### Luciferase reporter assay

A549 and SPC-A1 cells (2.0 × 10^4^) grown in a 96-well plates were co-transfected with 150 ng of LINC02418-WT or LINC02418-Mut reporters (Sangon Biotech, Shanghai, China) and miR-4677-3p mimic or NC mimics into LAD cells by use of Lipofectamine 2000 (Invitrogen, Carlsbad, California, USA). KNL1-WT or KNL1-Mut reporters (Sangon Biotech, Shanghai, China) and miR-4677-3p mimics or NC mimics were also co-transfected into indicated LAD cells. After transfection for 2 days, the luciferase activity normalized to Renilla luciferase activity was examined by luciferase reporter assay system (Promega, Madison WI).

### RNA immunoprecipitation (RIP) assay

RIP assay was conducted utilizing the EZ-Magna RIP kit (Millipore, Billerica, MA) according to the manufacturer’s protocol. A549 and SPCA1 cells at 80–90% confluence were obtained, and then lysed in complete RIP lysis buffer. Cell extract was processed at 4 °C for about 6 h with RIP buffer which contained human Ago2 antibody or control IgG (Millipore) coated magnetic beads. After beads washed, the RNA complexes were cultured with Proteinase K to digest proteins. RNA concentration was measured though employing a NanoDrop spectrophotometer (Thermo Scientific), with the quality assessed by a bioanalyser (Agilent, Santa Clara, CA). Finally, the immunoprecipitated RNAs were purified and analysed by RT-qPCR.

### RNA pull-down assay

RNA pull-down assay was used to detect the probable interaction among miR-4677-3p, LINC02418 and KNL1. MiR-4677-3p was biotinylated to be miR-4677-3p biotin probe by GenePharma Company (Shanghai, China). MiR-4677-3p biotin probe and miR-4677-3p no-biotin probe were added into the lysates of A549 and SPC-A1 cells. After 48 h incubation, Dynabeads M-280 Streptavidin (Invitrogen, CA) were put into above mixture. Two hours later, RNA in the pulled down complexes was examined using RT-qPCR analysis after purification.

### Statistical analysis

Each assay was implemented in triplicate and data were exhibited as mean ± standard deviation (SD). Based on SPSS for Windows, Version 14.0. (SPSS Inc., Chicago), student’s t-test was employed to compare differences between two groups, while one-way analysis of variance (ANOVA) was applied for the comparisons among no less than two groups. *P* < 0.05 was deemed as statistically significant.

## Results

### The biological function of LINC02418 in LAD

**Fig. 1:**
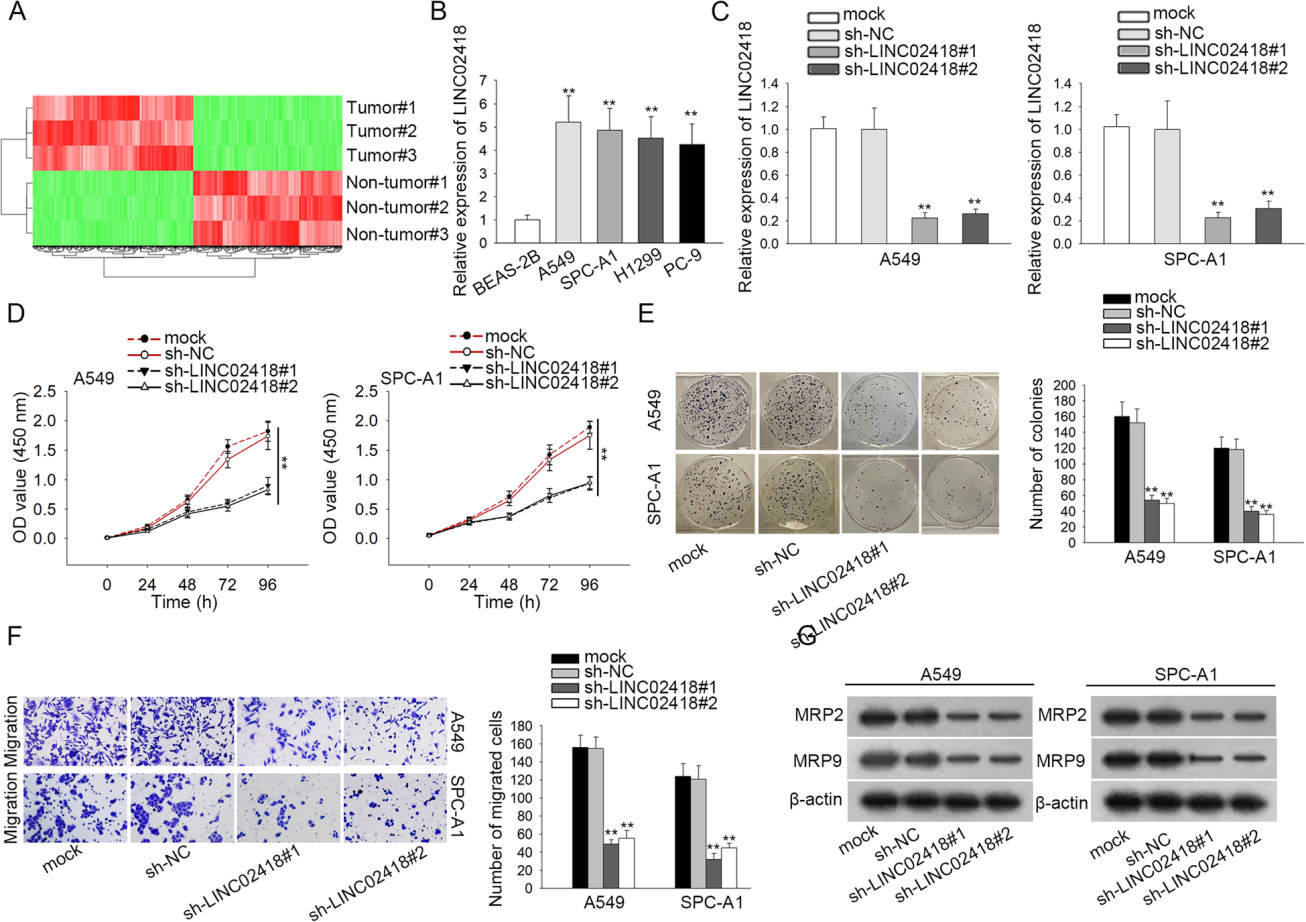
The biological function of LINC02418 in LAD. **a** Heatmap showing highly expressed LINC02418 in LAD. **b** LINC02418 expression in LAD cell lines and normal pulmonary epithelial cell line was examined by RT-qPCR. **c** The transfection efficiency of sh-LINC02418#1 and sh-LINC02418#2 in A549 and SPC-A1 cells were measured by RT-qPCR. **d** CCK-8 assay was performed to assess cell proliferation of A549 and SPC-A1 cells after the knockdown of LINC02418. **e** Colony formation assay was carried out to detect the proliferation of A549 and SPC-A1 cells in response to LINC02418 knockdown. **f** Transwell assay was applied to access cell migration in A549 and SPC-A1 cells after downregulation LINC02418. **g** Western blot was used to examine the level of migration-related proteins after knocking down LINC02418. ^**^P < 0.01

To identify the molecular mechanisms underlying LAD development, we first examined the differentially expressed lncRNAs in LAD samples relative to paired non-tumor ones. The upregulated lncRNAs in LAD tissues in comparison with adjacent normal tissues were displayed using a heatmap (Fig. 1a). Then, the top ten upregulated lncRNAs in LAD tissues were further analyzed in LAD cell lines via qRT-PCR. Results manifested that compared to the normal BEAS-2B cells, LINC02418 was expressed higher in four LAD cell lines (Fig. 1b; ^**^P < 0.01). However, other 9 lncRNAs were not significantly differential expressed in the four kinds of LAD cells relative to control cells (Figure S1A; ^*^P < 0.05, ^**^P < 0.01, n.s. was no significance). Thus, we chose LINC02418 as the research object in subsequent experiments. A549 and SPC-A1 cells possessing highest LINC02418 level were used for loss-of function assays. To probe the biological role of LINC02418, LINC02418 was knocked down in A549 and SPC-A1 cells by transfection with sh-LINC02418#1/2, with sh-NC-transfected cells as the scramble control. The results showed that knockdown of LINC02418 resulted in an obvious decline in LINC02418 expression compared with control group (Fig. 1c; ^*^P < 0.05). Additionally, CCK-8 and colony formation assays indicated that LINC02418 absence inhibited the proliferation of these two LAD cells (Fig. 1d-e; ^**^P < 0.01). TUNEL assay illustrated that cell apoptosis was promoted by LINC02418 depletion (Figure S2A; ^**^P < 0.01). As shown in Fig. 1f (^**^P < 0.01) and S2B (^**^P < 0.01), the migration and invasion of sh-LINC02418#1/2-transfected cells were notably blocked in contrast to that in sh-NC group, suggesting that LINC02418 silencing weakened LAD cell migration and invasion abilities. Furthermore, western blot analyses displayed that silencing LINC02418 suppressed the expression of migration-related proteins (MRP2 and MRP9), which indicated that LINC02418 depletion could inhibit cell migration (Fig. 1g). Further, when downregulating LINC02418, the levels of Bax and E-cadherin were augmented while Bcl-2 and N-cadherin expression was declined (Figure S2C). In total, LINC02418 is upregulated and LINC02418 knockdown suppresses cell proliferation and motility in LAD.

### The specific role of miR-4677-3p in LAD

**Fig. 2:**
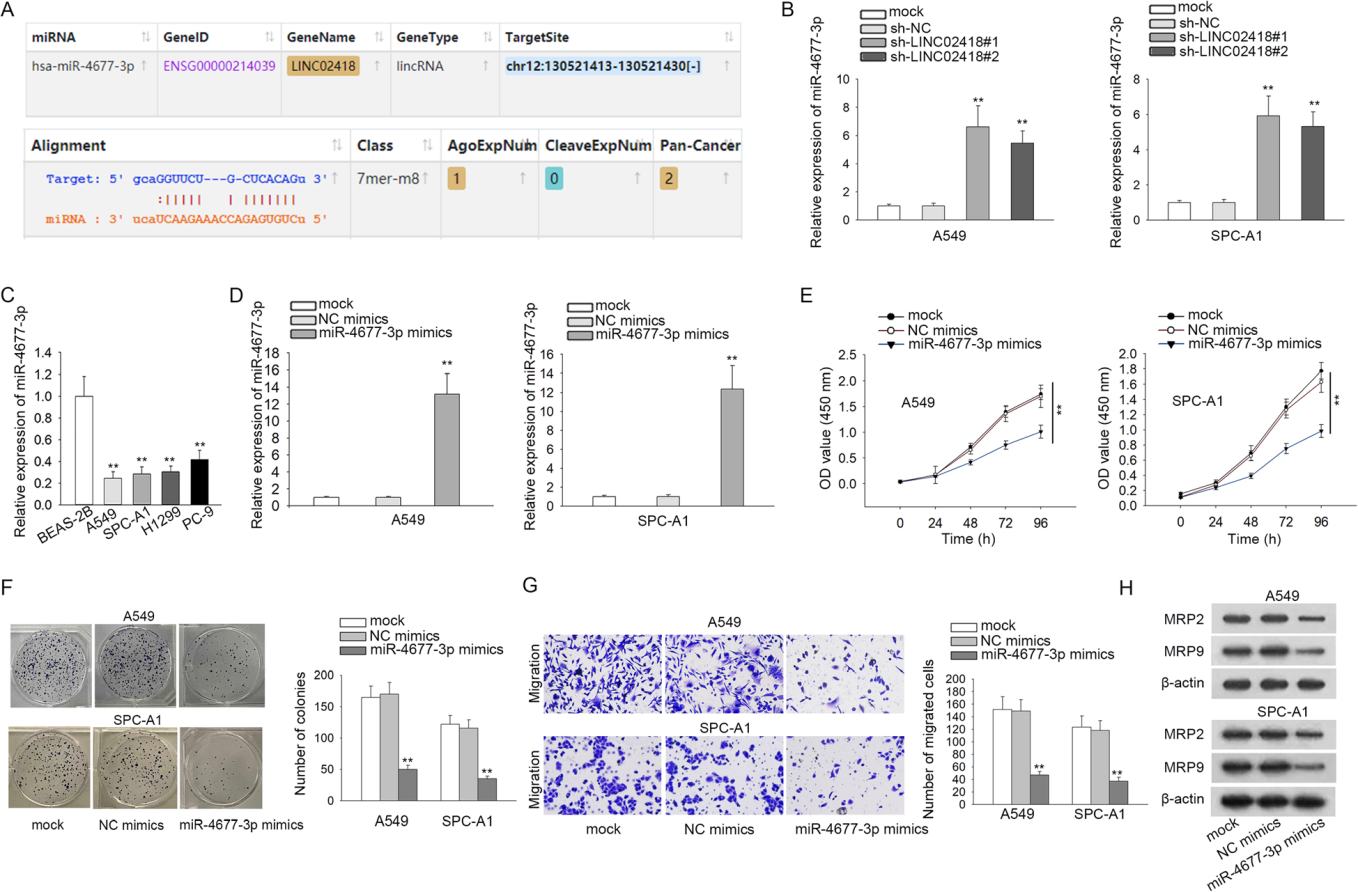
The specific role of miR-4677-3p in LAD. (**a**) Potential binding targets for LINC02418 were predicted by bioinformatics analysis. (**b**) Relative expression of miR-4677-3p influenced by LINC02418 knockdown was detected via RT-qPCR. (**c**) Relative expression of miR-4677-3p in LAD cell lines and normal pulmonary epithelial cell line was examined by RT-qPCR. (**d**) The transfection efficiency of miR-4677-3p mimics was checked by RT-qPCR. (**e**) CCK-8 assay was carried out to test cell proliferation when A549 and SPC-A1 cells were treated with miR-4677-3p mimics. (**f**) Colony formation assay was used to detect cell proliferation in miR-4677-3p mimics-transfected A549 and SPC-A1 cells (**g**) Transwell assay was performed to examine the migration of A549 and SPC-A1 cells treated with miR-4677-3p mimics. (**h**) Western blot was conducted to access the level of migration-related proteins in A549 and SPC-A1 cells when overexpressing miR-4677-3p. ^**^P < 0.01

Thereafter, bioinformatics analyses were employed to predict the miRNAs that could possibly bind with LINC02418. As a result, miR-4677-3p was then identified from starBase (Fig. 2a). In order to investigate whether LINC02418 could regulate miR-4677-3p expression, RT-qPCR analysis was performed. Interestingly, an observable increase in the expression of miR-4677-3p was observed in LAD cells with LINC02418 deficiency (Fig. 2b; ^**^P < 0.01). Additionally, miR-4677-3p was found to be with a low expression trend in LAD cell lines relative to BEAS-2B cells (Fig. 2c; ^**^P < 0.01). Subsequently, we observed a significantly heightened level of miR-4677-3p in miR-4677-3p mimics group compared with NC mimics group (Fig. 2d; ^**^P < 0.01). Moreover, miR-4677-3p mimics suppressed cell proliferation in two LAD cells (Fig. 2e-f; ^**^P < 0.01). Furthermore, overexpression of miR-4677-3p notably impaired LAD cell migration capability (Fig. 2g; ^**^P < 0.01). Moreover, as displayed in Fig. 2h, miR-4677-3p upregulation decreased the expression of migration-related proteins (MRP2 and MRP9). Taken together, miR-4677-3p is downregulated and serves a tumor-restraining part in LAD.

### KNL1 is a downstream target gene of miR-4677-3p

**Fig. 3:**
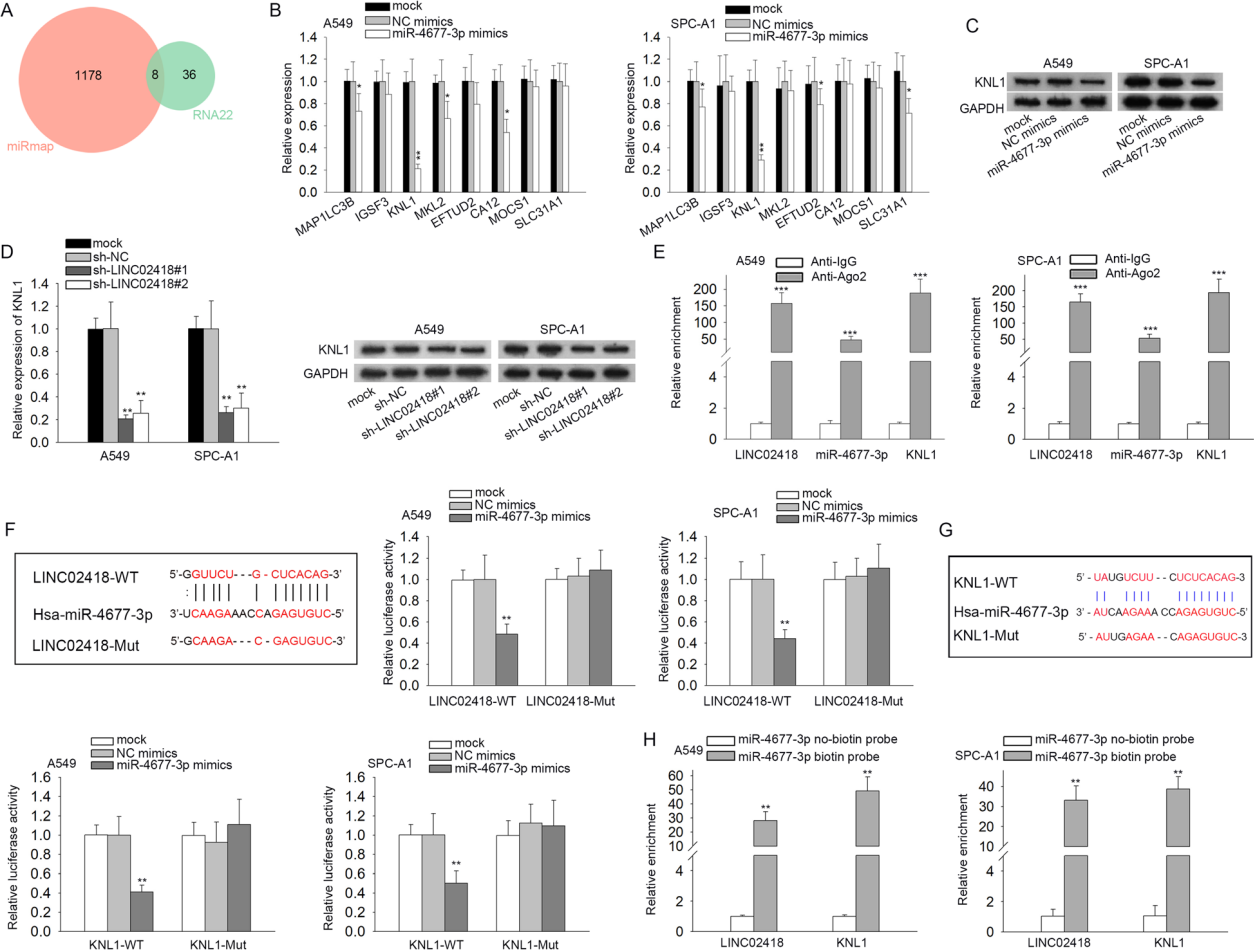
KNL1 is a downstream target gene of miR-4677-3p. **a** RNA22 and miRmap were used to research the potential target genes for miR-4677-3p. **b** The expression of the indicated mRNAs in miR-4677-3p mimic-transfected cells were tested using RT-qPCR. **c** The expression of KNL1 protein was detected by western blot when upregulating miR-4677-3p. **d** The mRNA and protein levels of KNL1 were examined by RT-qPCR and western blot analyses after downregulating LINC02418. **e** The enrichment of LINC02418, miR-4677-3p and KNL1 in RISC complex was determined by RIP assay. **f** The binding site between miR-4677-3p and LINC02418 was predicted by starBase (left). Luciferase reporter assay measured the luciferase activities of LINC02418-WT and LINC02418-Mut (right). **g** The binding site between miR-4677-3p and KNL1 was predicted by starBase (upper). Luciferase reporter assay measured the luciferase activities of KNL1-WT and KNL1-Mut. **h** RNA pull down assay examined the enrichment of LINC02418 and KNL1 in miR-4677-3p biotin probe group. miR-4677-3p no-biotin probe served as negative control. ^*^P < 0.05, ^**^P < 0.01, ^***^P < 0.001

Based on research over the past decades, miRNAs are known to have a vital impact on cancer progression by directly modulating the expression of its target genes [24–26]. Herein, 8 candidates (MAP 1LC3B, IGSF3, KNL1, MKL2, EFTUD2, CA12, MOCS1 and SLC31A1) were identified as the potential targets of miR-4677-3p following analyses of the RNA22 and miRmap databases (Fig. 3a). RT-qPCR was then employed to assess the influence of miR-4677-3p on the expression of these genes in A549 and SPC-A1 cells. Results indicated that only KNL1 expression was notably reduced by miR-4677-3p mimics compared with that in NC mimics control group in both the two LAD cells, while the level of other genes not (Fig. 3b; ^*^P < 0.05, ^**^P < 0.01). Therefore, KNL1 was selected to carry out the following assays. As illustrated in Fig. 3c, the level of KNL1 protein was also decreased by miR-4677-3p mimics. Besides, both mRNA and protein expressions of KNL1 were also hampered in face of LINC02418 knockdown (Fig. 3d; ^**^P < 0.01). In addition, LINC02418, miR-4677-3p and KNL1 were all concentrated in anti-Ago2 group instead of anti-IgG group (Fig. 3e; ^***^P < 0.001). Furthermore, the binding sequences between miR-4677-3p and LINC02418 were obtained from starBase (Fig. 3f, left). Then we found that the luciferase activity of pmirGLO-LINC02418-WT was reduced by miR-4677-3p mimics whereas that of pmirGLO-LINC02418-Mut showed no obvious alteration between miR-4677-3p mimic group and NC mimic group (Fig. 3f, right; ^**^P < 0.01). Similarly, starBase predicted the sequences of miR-4677-3p and KNL1 where the binding occurred (Fig. 3g, upper). The luciferase activity of pmirGLO-KNL1-WT was also lowered by enhanced miR-4677-3p while that of pmirGLO-KNL1-Mut wasn’t affected (Fig. 3g, lower; ^**^P < 0.01). Moreover, the results of RNA pull-down assay testified that both LINC02418 and KNL1 were enriched in miR-4677-3p biotin probe group (Fig. 3h; ^**^P < 0.01). In sum, KNL1 is the downstream molecule of miR-4677-3p in LAD.

### LINC02418 promotes the malignant phenotypes of LAD cells through miR-4677-3p/KNL1 signaling

**Fig. 4:**
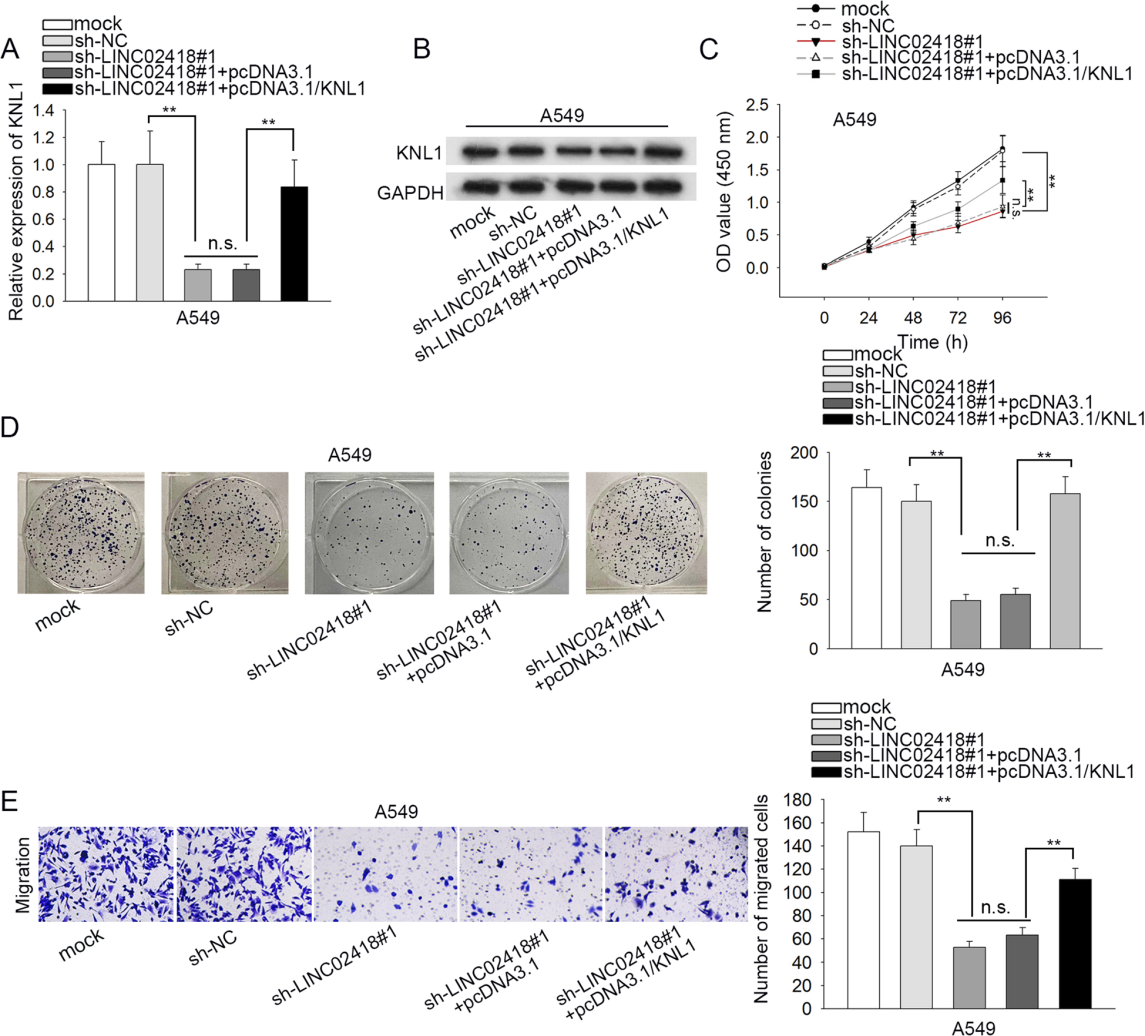
LINC02418 may promote malignancy in LAD by targeting miR-4677-3p/KNL1 axis. **a**-**b** The mRNA and protein expressions of KNL1 in differently transfected groups (were estimated by RT-qPCR and western blot. **c**-**d** Cell proliferation in differently transfected groups was evaluated by CCK-8 and colony formation assays. **e** Cell migration in differently transfected groups was detected by transwell assay. ^**^P < 0.01. n.s. means no significance

Subsequently, we planned to determine whether LINC02418 influenced LAD development through miR-4677-3p/KNL1 pathway. RT-qPCR and western blot analyses indicated that co-transfection of pcDNA3.1/KNL1 recovered LINC02418 depletion-lessened KNL1 expression in A549 cells (Fig. 4a-b; ^**^P < 0.01, n.s. was no significance). Then, it was verified that KNL1 upregulation reversed the hindering impact of LINC02418 deficiency on A549 cell proliferation (Fig. 4c-d; ^**^P < 0.01, n.s. was no significance). Additionally, the stimulated cell apoptosis induced by LINC02418 silencing was rescued by KNL1 overexpression (Figure S3A; ^**^P < 0.01, n.s. was no significance). Subsequently, it was confirmed that overexpression of KNL1 offset the obstructive function caused by LINC02418 depletion on A549 cell motility (Fig. 4e and S3B; ^**^P < 0.01, n.s. was no significance). Additionally, KNL1 upregulation neutralized the influence of LINC02418 depletion on the levels of Bax, Bcl-2, E-cadherin and N-cadherin (Figure S3C). These findings suggest that LINC02418 aggravates malignant behaviors in LAD via miR-4677-3p/KNL1 pathway.

## Discussion

Lung adenocarcinoma (LAD) is the most common subtype of non-small-cell lung cancer (NSCLC), accounting for the majority of diagnosed primary lung cancer cases, and also with low 5-year survival rate [27, 28]. In recent few decades, great progresses have been achieved in LAD treatment, including anti-PD-1/PD-L1 therapy and targeted therapy [29, 30]. In the meantime, treatment strategies for LAD have also improved [31–33]. Nonetheless, the survival rate of patients with LAD still remains poor. Hence, identifying effective targets is imperative for opening fresh strategies for LAD treatment.

Recently, a great amount of work has uncovered the veil of lncRNAs in multiple cancers, and a growing body of literature has delineated that lncRNAs are implicated in various malignancies, such as LAD, breast cancer and gastric cancer [12, 34, 35]. Nonetheless, whether LINC02418 works in LAD has not been revealed. Currently, we found the notable upregulation of LINC02418 in LAD, and the absence of LINC02418 suppressed LAD cell proliferation and motility, implying that LINC02418 promotes the malignancy in LAD.

MiRNAs are also defined as a fraction of ncRNAs with the length of 20–24 nucleotides [36, 37]. Previous research suggests that miRNAs exert their functions in diverse cancers [38, 39]. As an illustration, miR-4500 is downregulated and elicits an anti-cancer function through regulating HMGA2 expression in colorectal cancer [40]. MiR-431-5p targets UROC28 to influence the expression of EMT markers in hepatocellular carcinoma [41]. MiR-205 regulates E2F1 expression to promote the cisplatin sensitivity of glioma cells [42]. In LAD, miRNAs also play important roles, like miR-133 participates in LAD metastasis via targeting with FLOT2 [43]. MiR-629-3p inhibits SFTPC expression to facilitate cell proliferation and is associated with poor survival in LAD [44]. MiR-608 and miR-4513 greatly enhance the prognosis of LAD treated with EGFR-TKIs [45]. Present study showed the decreased expression of miR-4677-3p in LAD and revealed that miR-4677-3p overexpression suppressed cell proliferation and migration in LAD, highlighting miR-4677-3p as a cancer-suppressor in LAD.

Proteins translated from messenger RNAs (mRNAs) play critical roles in cancer. For instance, EGFR enhances the development of renal cancer [46]. YWHAZ serves as an oncogenic gene in cervical cancer [47]. Also, the oncogenic function of KNL1 has been validated in cancer. For example, miR-193b-3p silencing promotes cell proliferation in gastric cancer through upregulating the expression of KNL1 [48]. CeRNA hypothesis have been proposed and proven to be transcripts cross-regulated by competing certain miRNAs [49, 50]. To be specific, lncRNA and mRNA can competitively bind with the shared miRNA to modulate cancer progression. For instance, lncRNA HOXD-AS1 promotes liver cancer metastasis through sponging miR-130a-3p and targeting SOX4 [51]. LncRNA TUG1 facilitates the development of papillary thyroid cancer via miR-145/ZEB1 axis [52]. LncRNA-UCA1 exerts its oncogenic function in esophageal cancer through acting as the ceRNA of SOX4 [53]. Our study revealed that miR-4677-3p could bind with LINC02418 and KNL1, and KNL1 was the mediator downstream of LINC02418/miR-4677-3p signaling in LAD. Finally, rescue assays indicated that the inhibited LAD cell functions induced by LINC02418 silencing were counteracted by KNL1 overexpression.

Upregulation of LINC02418 in LAD tissue samples was identified by a microarray analysis, indicating the clinical potential of LINC02418 in LAD patients. This study didn’t elucidate the role of LINC02418 in clinical features or prognosis. Thus, we will investigate the clinical value of LINC02418 in LAD in future research. In conclusion, LINC02418 contributes to malignant phenotypes of LAD cells through sequestering miR-4677-3p to boost KNL1 level, throwing light on the molecular mechanism of LINC02418 in LAD, providing a novel target for LAD treatment.

## Conclusions

LINC02418 facilitates malignant cell behaviors in LAD via sponging miR-4677-3p to upregulate KNL1 expression.

## Supplementary information

**Additional file 1: Figure S1.** (A) Expression pattern of 9 lncRNAs in LAD cells and normal BEAS-2B cell was tested by RT-qPCR. ^*^P < 0.05, ^**^P < 0.01; n.s.: no significance.

**Additional file 2: Figure S2.** (A) TUNEL assay measured cell apoptosis in LINC02418 downregulated cells. (B) Transwell assay detected cell invasion when knocking down LINC02418. (C) Western blot tested expression of apoptosis- and EMT-related proteins in response to LINC02418 depletion. ^**^P < 0.01.

**Additional file 3: Figure S3.** (A) TUNEL assay measured cell apoptosis in differently transfected groups. (B) Transwell assay detected cell invasion in differently transfected groups. (C) Western blot tested expression of apoptosis- and EMT-related proteins in differently transfected groups. ^**^P < 0.01; n.s.: no significance.

**Additional file 4: Supplementary Information file 1.** The original, unprocessed gel images for western blot data in Figs. 1g, 2h, 3c, d, 4b, S2C and S3C.

## Data Availability

Relevant data and materials have been presented within the manuscript and additional files.

## Abbreviations

ANCR: Angelman syndrome chromosome region
ANOVA: Analysis of variance
CA12: Carbonic anhydrase 12
CCK-8: Cell counting kit-8
ceRNA: Competing endogenous RNA
DGCR5: DiGeorge syndrome critical region gene 5
DMEM: Dulbecco’s modified Eagle’s medium
DNMT1: DNA methyltransferase 1
E2F1: E2F transcription factor 1
EFTUD2: Elongation factor Tu GTP binding domain containing 2
EGFR: Epidermal growth factor receptor
EMT: Epithelial-mesenchymal transition
EZH2: Enhancer of zeste 2 polycomb repressive complex 2 subunit
FBS: Fetal bovine serum
GAS5: Growth arrest specific 5
HMGA2: High mobility group AT-hook 2
HOTAIR: HOX transcript antisense RNA
HOXD-AS1: HOXD antisense growth-associated long non-coding RNA
IGSF3: Immunoglobulin superfamily member 3
KNL1: Kinetochore scaffold 1
LAD: Lung adenocarcinoma
LINC02418: Long intergenic non-protein coding RNA 2418
lncRNAs: Long noncoding RNAs
MALAT1: Metastasis associated lung adenocarcinoma transcript 1
MAP 1 LC3B: Microtubule associated protein 1 light chain 3 beta
MIR31HG: MIR31 host gene
miRNAs: microRNAs
MKL2: Myocardin like 2
MOCS1: Molybdenum cofactor synthesis 1
n.s.: no significance
NEAT1: Nuclear paraspeckle assembly transcript 1
NSCLC: Non-small cell lung cancer
PVT1: Pvt1 oncogene
RIP: RNA immunoprecipitation
RIPA: Radioimmunoprecipitation assay
RT-qPCR: Real-time quantitative polymerase chain reaction
SD: Standard deviation
SLC31A1: Solute carrier family 31 member 1
SNHG1: Small nucleolar RNA host gene 1
SOX4: SRY-box transcription factor 4
TIMP2: TIMP metallopeptidase inhibitor 2
TLR4: Toll like receptor 4
TUG1: Taurine up-regulated 1
TUNEL: Terminal-deoxynucleoitidyl transferase mediated nick end labeling
UCA1: Urothelial cancer associated 1
YWHAZ: Tyrosine 3-monooxygenase/tryptophan 5-monooxygenase activation protein zeta
ZEB1: Zinc finger E-box binding homeobox 1
