# Supplementary figures and images for: LINC02418 promotes malignant behaviors in lung adenocarcinoma cells by sponging miR-4677-3p to upregulate KNL1 expression
Source: BMC Pulm Med. 2020 Aug 14;20:217. doi: 10.1186/s12890-020-01229-0 (PMC7427971; doi:10.1186/s12890-020-01229-0)

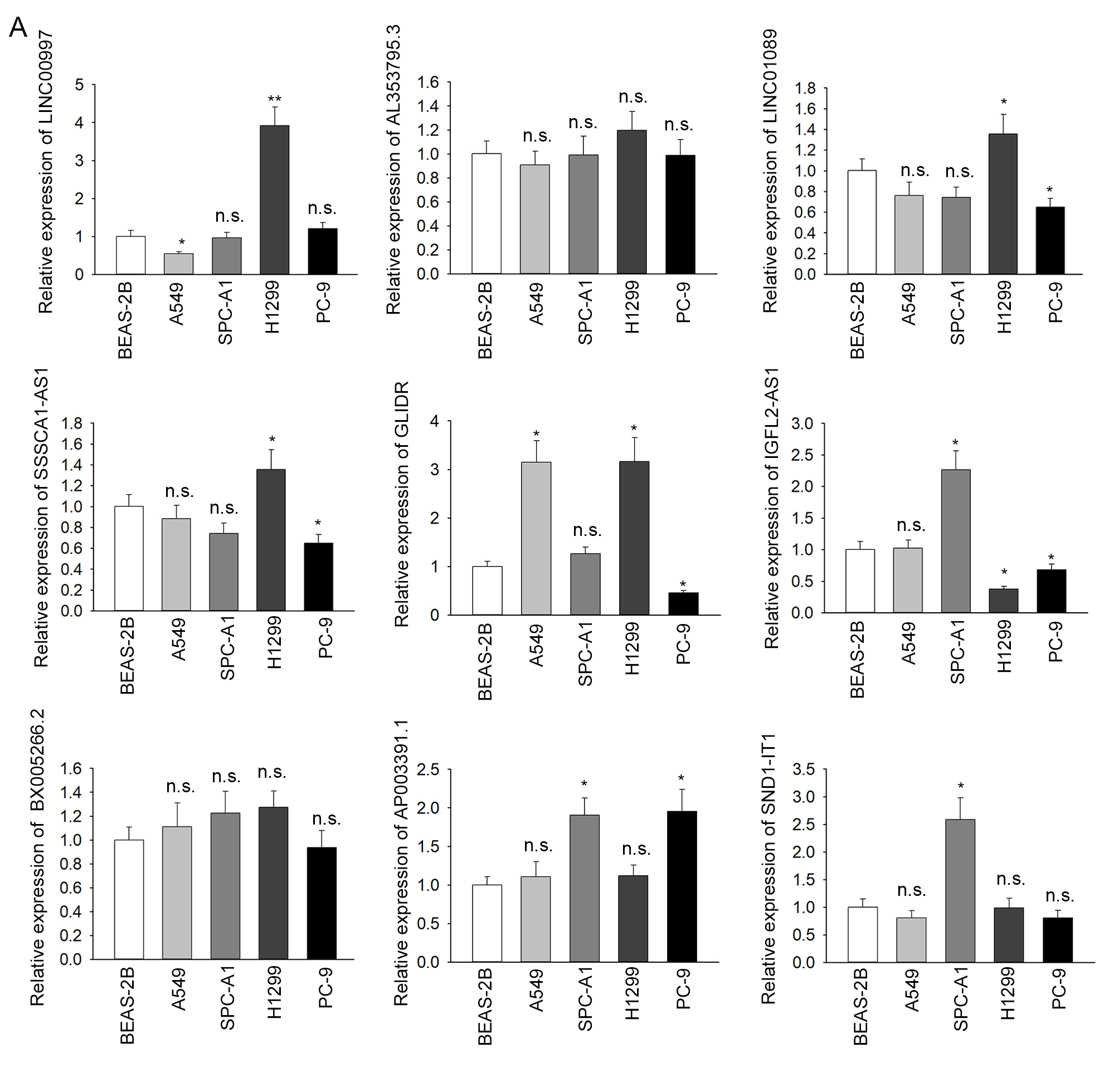

Supplement: Supplementary file 1 — Additional file 1: Figure S1. (A) Expression pattern of 9 lncRNAs in LAD cells and normal BEAS-2B cell was tested by RT-qPCR. *P < 0.05, **P < 0.01; n.s.: no significance. [file 12890_2020_1229_MOESM1_ESM.tif]

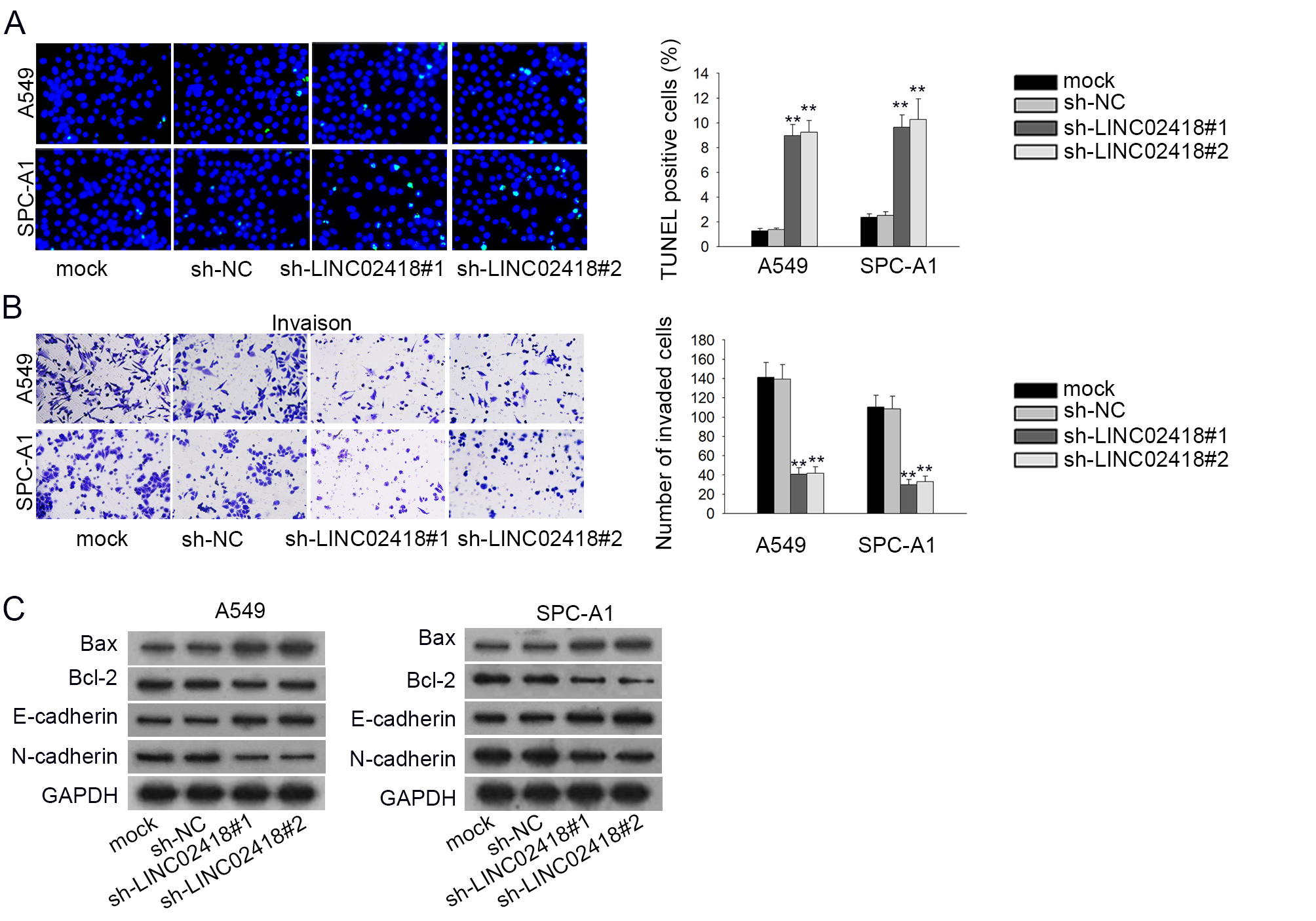

Supplement: Supplementary file 2 — Additional file 2: Figure S2. (A) TUNEL assay measured cell apoptosis in LINC02418 downregulated cells. (B) Transwell assay detected cell invasion when knocking down LINC02418. (C) Western blot tested expression of apoptosis- and EMT-related proteins in response to LINC02418 depletion. **P < 0.01. [file 12890_2020_1229_MOESM2_ESM.tif]

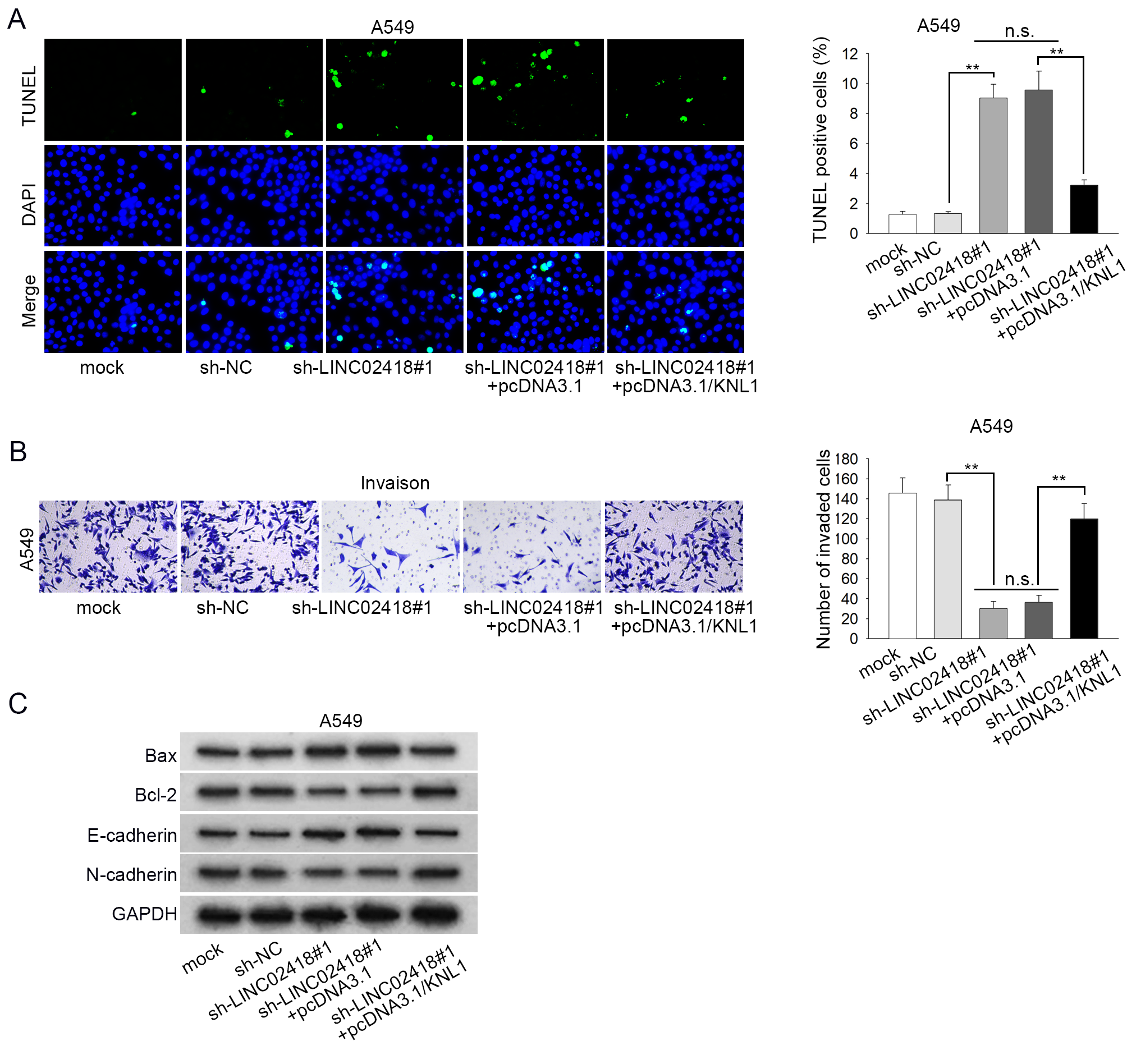

Supplement: Supplementary file 3 — Additional file 3: Figure S3. (A) TUNEL assay measured cell apoptosis in differently transfected groups. (B) Transwell assay detected cell invasion in differently transfected groups. (C) Western blot tested expression of apoptosis- and EMT-related proteins in differently transfected groups. **P < 0.01; n.s.: no significance. [file 12890_2020_1229_MOESM3_ESM.tif]

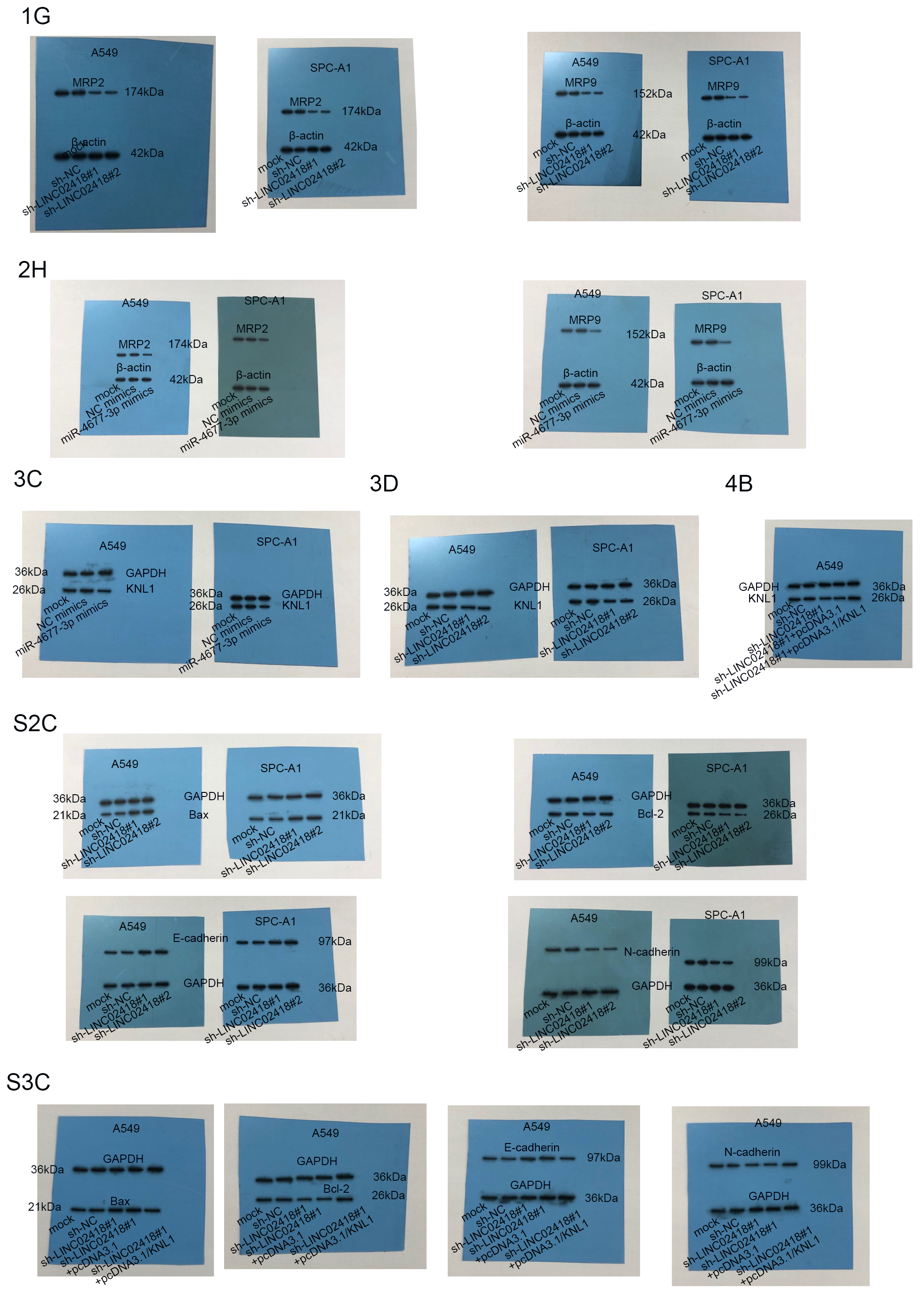

Supplement: Supplementary file 4 — Additional file 4: Supplementary Information file 1. The original, unprocessed gel images for western blot data in Figs. 1g, 2h, 3c, d, 4b, S2C and S3C. [file 12890_2020_1229_MOESM4_ESM.tif]
